# Advancing Public Health Surveillance in Child Care Centers: Stakeholder-Informed Redesign and User Satisfaction Evaluation of the MCRISP Network

**DOI:** 10.2196/60319

**Published:** 2024-09-24

**Authors:** William Gribbin, Peter Dejonge, Jakob Rodseth, Andrew Hashikawa

**Affiliations:** 1 Indiana University School of Medicine, 340 West 10th Street, Indianapolis, IN, United States, 1 317-274-8157; 2 School of Public Health, University of Michigan, Ann Arbor, MI, United States; 3 Department of Emergency Medicine, Michigan Medicine, Ann Arbor, MI, United States

**Keywords:** public health, disease surveillance, data collection, dashboard, child care, child, children, care center, user satisfaction, ill, illness, transmission, tracking, tracker, COVID-19, SARS-CoV-2, pandemic, disease monitoring, technology, respiratory, gastrointestinal, user-centered design, infectious disease, visualization

## Abstract

Leveraging user feedback, we redesigned a novel disease monitoring utility to allow for bidirectional data flow and in this letter offer insights into that process as well as lessons learned.

## Introduction

Child care centers are important hubs for monitoring respiratory and gastrointestinal illness transmission [1,2]. The Michigan Child Care Related Infection Surveillance Program (MCRISP) is a free website that empowers ~25 regional child care centers to submit illness reports and leverages that data to provide public health illness surveillance locally [3]. MCRISP has demonstrated functionality for sentinel reporting for outbreaks [4,5]. We previously gathered insights from child care providers (CCPs) on how to enhance MCRISP [6]. CCPs called for improvement in multidirectional data flow, enhanced data visualization, and fortified data security measures. We present an evaluation of our newly redesigned MCRISP 2.0, which was informed by feedback from our prior work. We include insights into the redesign process, users’ responses to the updated design, an overview of the technologies implemented, and user reactions to these changes.

## Methods

### Design Changes

**Figure 1.**
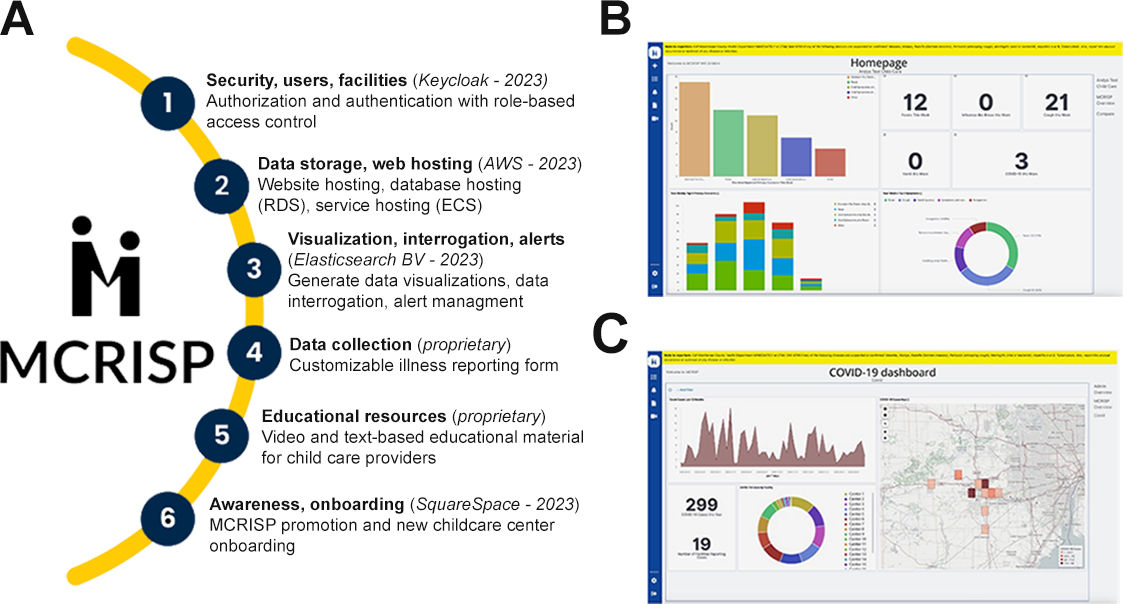
(A) Features implemented in the Michigan Child Care Related Infection Surveillance Program (MCRISP) website, technologies used to create those features, and the main use cases. (B) Child care provider homepage displays a customized visualization. (C) COVID-19 dashboard includes a 12-month weekly case count, pie chart, numerical element showing the total number of cases and facilities reporting cases, and a heat map of cases. AWS: Amazon Web Services; ECS: Elastic Container Service; RDS: Relational Database Service.

We redesigned MCRISP 1.0 by incorporating feedback from our prior work with CCPs to identify areas for improvement [6]. Multiple technologies were leveraged in the redesign process (Figure 1A). CCPs had a jointly developed custom health dashboard for their center and region (Figure 1B). More advanced dashboards were made for users working in public health and were easily developed with the new enhancements (Figure 1C).

### Setting and Data Analysis

MCRISP currently only enrolls child care centers located in Washtenaw County, Michigan. We invited, by email, 66 CCPs who had used MCRISP versions 1.0 or 2.0 to fill out a survey designed to assess their use and reaction to the new redesign (Multimedia Appendix 1). In total, 24 CCPs responded; the analysis includes the 19 complete responses. Data were compared using a 2-tailed paired-sample *t* test after confirming that the data did not violate normality. The α level was set at <.05.

### Ethical Considerations

This work was reviewed by the University of Michigan institutional review board and found to be exempt from classification as human subjects research.

## Results

### Inclusion

Of 66 past and present CCP MCRISP users, 24 (36%) responded, with 19 fully completing the questionnaire. Of those who completed questionnaires, 13 (68%) had experience using both versions of MCRISP; 5 respondents only used MCRISP 2.0 and 1 had only used MCRISP 1.0.

### Response to New Features

Among respondents who completed the survey and who were familiar with MCRISP 2.0 (18/19), we queried about CCP use patterns and experience with its features. Notably, 100% of respondents submitted illness reports at least weekly, with 61% (11/18) submitting reports multiple times per week. Over 83% (15/18) of respondents indicated they somewhat or strongly agreed that automated weekly email summaries were helpful. Regarding engagement with educational documents and video content, 67% (12/18) and 39% (7/18) of respondents used them at least monthly, respectively, with 50% (9/18) of respondents sharing resources with parents and 78% (14/18) saying they viewed illness graphics at least monthly.

### Comparing MCRISP 1.0 and 2.0

Among respondents familiar with both MCRISP 1.0 and 2.0 (13/19; 68%), respondents were likely to more strongly agree with statements about the positive aspects of MCRISP 2.0 than MCRISP 1.0, though this positive trend was statistically nonsignificant (Table 1).

**Table 1.** There were no statistically significant differences in individual user (n=13) agreement with various queried aspects of the MCRISP user experience between versions 1.0 and 2.0. Respondents answered the following question on a 5-point scale: “Please indicate your level of agreement with the following aspects of the [X] version of MCRISP: [Y]” where the X was replaced with MCRISP 1.0 or 2.0, and the Y was replaced with the questions listed below; answers were rated as 1=strongly disagree, 2=somewhat disagree, 3=neither agree or disagree, 4=somewhat agree, and 5=strongly agree. Mean ∆ refers to the mean per-user change in agreement level from the MCRISP 1.0 question versus the MCRISP 2.0 question. A positive mean ∆ value indicates users more strongly agreed with the question when it was asked about MCRISP 2.0 than MCRISP 1.0, whereas a negative value indicates the opposite. Paired-sample *t* tests (2-tailed) were conducted (n=13; *df*=1).

Question	Score for MCRISP 1.0, mean (SD)	Score for MCRISP 2.0, mean (SD)	Mean ∆	*t* test (*df*)	*P* value
“The [website] is/was easy to use”	3.69 (1.32)	4.08 (1.04)	+0.39	1.05 (1)	.32
“I use/used the graphical data on the [website] to make informed decisions at our center”	3.00 (1.00)	3.31 (1.38)	+0.31	1.29 (1)	.22
“I find/found it easy to submit illness reports”	4.08 (1.38)	4.15 (1.07)	+0.08	0.04 (1)	.84
“I felt connected to the child care community when I use/used [website]”	3.46 (1.13)	3.38 (1.33)	-0.08	0.13 (1)	.72
“I use/used [website] frequently (multiple times per week)”	3.23 (1.17)	3.85 (1.28)	+0.63	3.46 (1)	.09

## Discussion

### Principal Findings

MCRISP is unique as a community-based biosurveillance platform that not only integrates suggestions from CCP user focus groups but is also designed to facilitate a bidirectional flow of information. This approach is critical as community engagement, transparency, and inclusivity are paramount in research and public health initiatives. MCRISP is unique not only among other US-based surveillance systems but also among international counterparts such as the KIzSS network (Dutch abbreviation for National Multicenter, Day Care–Based Sentinel Surveillance Network for Infectious Diseases) in the Netherlands [7]. KIzSS is a government-sponsored, national surveillance network that releases reports annually. It lacks real-time data access for stakeholders, unlike MCRISP, which supports continuous, multidirectional data flow through a partnership between public health, researchers, and CCPs. MCRISP is reliant on CCPs as partners in maintaining the network’s surveillance capabilities; therefore, introducing changes risks causing disengagement among users who may find the new technology too difficult to use or fit into their workflow [8,9]. Our stakeholder-informed approach yielded improved system capabilities and maintained user engagement. To replicate a comparable public health–centered child care surveillance system, we recommend using focus groups and an iterative design philosophy to ensure stakeholders remain motivated to contribute data.

### Limitations

MCRISP currently operates within 1 county and does not include smaller or home-based child care facilities, which may limit its generalizability.

### Conclusions

MCRISP represents a paradigm shift in community disease surveillance by leveraging the child care system. MCRISP can serve as a guide for developing a stakeholder-focused approach for public health surveillance initiatives.

## Supplementary material

10.2196/60319 Multimedia Appendix 1 Questionnaire provided to child care providers.

## Abbreviations

CCP: child care provider
KIzSS: National Multicenter, Day Care–Based Sentinel Surveillance Network for Infectious Diseases [Dutch abbreviation]
MCRISP: Michigan Child Care Related Infection Surveillance Program
