# Supplementary material for: Advancing Public Health Surveillance in Child Care Centers: Stakeholder-Informed Redesign and User Satisfaction Evaluation of the MCRISP Network
Source: JMIR Public Health Surveill. 2024 Sep 24;10:e60319. doi: 10.2196/60319 (PMC11443984; doi:10.2196/60319)
Supplement: Multimedia Appendix 1 [file publichealth-v10-e60319-s001.docx]

MCRISP 2.0 User Feedback (Supplemental Form 1)

Start of Block: Default Question Block

Your feedback on MCRISP is valuable to us and helps us improve the quality of your experience. We appreciate you taking time to fill out this 3-minute survey.

| 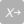 |
| --- |

Q1 What is the average daily enrollment of your program (number of children)?

- Less than 12 (1)
- 13-20 (2)
- 21-50 (3)
- 51-100 (4)
- 101-200 (5)
- 201+ (6)

| 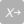 |
| --- |

Q2 Did you, the respondent to this survey, have experience using MCRISP before it was updated (known as sickchildcare.org)?

- Yes
- No

| 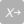 |
| --- |

Q3 Do you have experience using the current version of MCRISP (MCRISP 2.0)?

- Yes
- No

Display This Question:

If Did you, the respondent to this survey, have experience using MCRISP before it was updated (known... = Yes

|  |
| --- |

Q4 Please indicate your level of agreement with the following aspects of the old version of MCRISP (sickchildcare.org).

|  | Strongly disagree (1) | Somewhat disagree (2) | Neither agree nor disagree (3) | Somewhat agree (4) | Strongly agree (5) |
| --- | --- | --- | --- | --- | --- |
| The old website (sickchildcare.org) was easy to use (Q4_1) |  |  |  |  |  |
| I used the graphical data on the old website to make informed decisions at our center (Q4_2) |  |  |  |  |  |
| I found it easy to submit illness reports (Q4_3) |  |  |  |  |  |
| I felt connected to the child care community when I used sickchildcare.org (Q4_4) |  |  |  |  |  |
| I used sickchildcare.org frequently (multiple times per week) (Q4_5) |  |  |  |  |  |

Display This Question:

If Do you have experience using the current version of MCRISP (MCRISP 2.0)? = Yes

Q5 Please indicate your level of agreement with the following aspects of the current version of MCRISP (MCRISP.org).
 

|  | Strongly disagree (1) | Somewhat disagree (2) | Neither agree nor disagree (3) | Somewhat agree (4) | Strongly agree (5) |
| --- | --- | --- | --- | --- | --- |
| The current website (MCRISP.org) is easy to use (Q5_1) |  |  |  |  |  |
| I used the graphical data on the current website to make informed decisions at our center (Q5_2) |  |  |  |  |  |
| I find it easy to submit illness reports (Q5_3) |  |  |  |  |  |
| I feel connected to the child care community when I use MCRISP.org (Q5_4) |  |  |  |  |  |
| I use MCRISP.org frequently (multiple times per week) (Q5_5) |  |  |  |  |  |
| The weekly automated illness email summaries sent to me are helpful (Q5_6) |  |  |  |  |  |
| Our center shares educational resources from the website with parents (Q5_7) |  |  |  |  |  |

Display This Question:

If Do you have experience using the current version of MCRISP (MCRISP 2.0)? = Yes

| 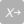 |
| --- |

Q6 How often, on average, do you use the following features of the current version MCRISP (2.0)? Please, use your best estimate.

|  | Never (1) | Once a month or less (2) | Once a week (3) | Multiple times a week (4) |
| --- | --- | --- | --- | --- |
| View illness graphs for my center (Q6_1) |  |  |  |  |
| View illness graphs of centers in my area (Q6_2) |  |  |  |  |
| View illness graphs our center to all other centers (Q6_3) |  |  |  |  |
| Submit illness reports (Q6_4) |  |  |  |  |
| Subscribe to illness alerts (Q6_5) |  |  |  |  |
| Use educational resource handouts to give to parents (Q6_6) |  |  |  |  |
| Watch the video resources (Q6_7) |  |  |  |  |
| Review our previously submitted illness reports (Q6_8) |  |  |  |  |

Display This Question:

If Do you have experience using the current version of MCRISP (MCRISP 2.0)? = Yes

| 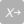 |
| --- |

Q7 How would you rate your experience using the following features of the current version of MCRISP (2.0)?

|  | Very Poor (1) | Below Average (2) | Average (3) | Above Average (4) | Excellent (5) |
| --- | --- | --- | --- | --- | --- |
| Viewing data displays (graphs, figures, case numbers) (Q7_1) |  |  |  |  |  |
| Submitting illness reports (Q7_2) |  |  |  |  |  |
| Modifying previously submitted illness reports (Q7_3) |  |  |  |  |  |
| Using the educational handout resources on the site (Q7_4) |  |  |  |  |  |
| Using the educational video resources on the site (Q7_5) |  |  |  |  |  |

Q8 What other features, if any, would you like to see implemented next in MCRISP 3.0?

________________________________________________________________

________________________________________________________________

________________________________________________________________

________________________________________________________________

________________________________________________________________

Q9 Does MCRISP 2.0 provide value for your center? If yes, please explain.

________________________________________________________________

________________________________________________________________

________________________________________________________________

________________________________________________________________

________________________________________________________________

Q10 What else would you like the creators of MCRISP creators to know?

________________________________________________________________

________________________________________________________________

________________________________________________________________

________________________________________________________________

________________________________________________________________

End of Block: Default Question Block
